# Liver fluke and schistosome cross-infection risk between livestock and wild mammals in Western Uganda, a One Health approach

**DOI:** 10.1016/j.ijppaw.2024.101022

**Published:** 2024-11-19

**Authors:** Daisy Namirembe, Tine Huyse, Rapheal Wangalwa, Julius Tumusiime, Casim Umba Tolo

**Affiliations:** a Department of Biology, Mbarara University of Science and Technology, Uganda; b Department of Biology, Royal Museum for Central Africa, Belgium

**Keywords:** *Schistosoma bovis*, *Schistosoma mattheei*, *Fasciola*, Livestock-wild mammal interaction, Cross-infection, Zoonosis, One health, Lake Albert, Uganda

## Abstract

Trematodiases strongly reduce the welfare of humans and animals causing a great decline in health and productivity. Insufficient data on the extent of trematode infection in definitive hosts and associated risk factors remain a great threat to its control. A cross-sectional study was conducted to establish the; prevalence of liver flukes and schistosomes in livestock and wild mammals and the socio-ecological risk factors associated with their spread. Fresh dung samples were collected opportunistically (n = 865) and examined using formal ether sedimentation and microscopy for parasite eggs. Twelve abattoir visits were conducted to examine the livers of animals killed for mature flukes. Key informants (n = 110) including farmers, butchers, game rangers, and herders were interviewed to document the socio-ecological risk factors. In the abattoirs, 57.1%(CI 0.422–0.712) of cattle were infected with *Fasciola* flukes and not sheep and goats. Cattle dung had the highest prevalence (56% CI 0.518–0.604) of *Fasciola* eggs, followed by sheep (50%, CI 0.319–0.681) and goats (28.2%, CI 0.218–0.353). Among wild mammals, hippos' dung (66%; 95% CI 0.53–0.777) had the highest prevalence of *Fasciola* followed by warthogs (8%; 95% CI 0.002–0.385) and baboons (6.7%; CI 0.002–0.319). No *Fasciola* eggs were observed in elephant dung (n = 21) and monkeys (n = 2). *Schistosoma bovis* was found in cattle dung from Mpeefu (2.6%; 95% CI 0.007–0.066) and Ndaiga (4.3%; 95% CI 0.022–0.075) while *S. mattheei* in goats’ (1.4%; 95% CI 0.00–0.075) and cattle (0.39%; 95% CI 0.00–0.021) dung samples from Ndaiga. Key informants had moderate knowledge of fasciolosis (62.7%), highest among butchers (89.7%), and lowest among herders (31.8%). Only veterinary officers knew about schistosomiasis in animals. Free-range grazing and unsafe water sources for livestock, shared with wild animals, were the risky practices by most farmers (66–100%). *Fasciola* was prevalent in livestock and wild mammals, while *Schistosoma* in cattle and goats.

## Highlights

• *Livestock*-wildlife interfaces are high-risk zones for the spread of zoonotic parasites.
• *Schistosoma bovis* and *S. mattheei* are existent in livestock at the shores of Lake Albert in Uganda.
• *Fasciola* parasites can infect both livestock and wild mammals.
• The One Health Approach is paramount when tackling human, animal and environmental health because they are interconnected.

## Graphical abstract

**Figure:**
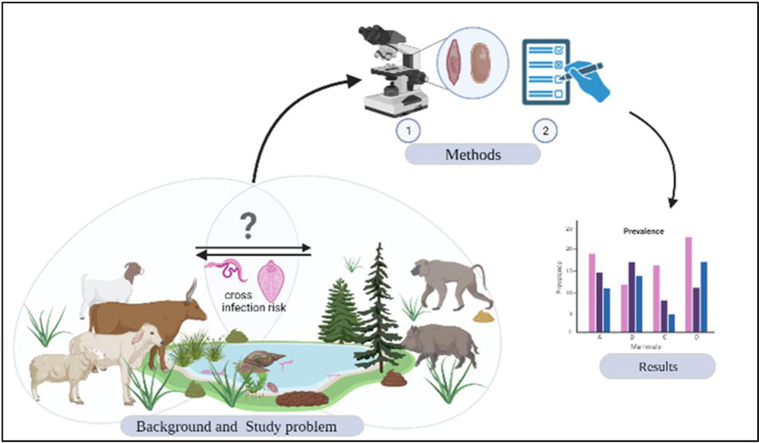

## 1 Introduction

Zoonotic diseases are naturally occurring infections that can be transmitted between animals and humans. They represent approximately 60% of emerging infectious diseases, of which 75% have originated from animals, and 70 million people are at risk (Richard Stauffer and Madsen, 2018). Fasciolosis and schistosomiasis are caused by *Fasciola* and *Schistosoma* flatworms, respectively, which are snail-borne trematodes infecting both animals and humans (Nelwan, 2019a; Nelwan, 2019b; Gryseels et al., 2006). The parasites present a unique life cycle, undergoing sexual reproduction in vertebrate definitive hosts and asexual reproduction in snail intermediate hosts. Viable eggs of the *Fasciola* and *Schistosoma* parasites are expelled in feces and or urine, respectively, by infected final hosts, then hatch into miracidia and enter the appropriate snail intermediate host, inside which they undergo several development stages (asexual reproduction), giving rise to the larval stage of cercariae. *Fasciola* cercariae attach to vegetation and encyst into metacercariae, ready to be ingested by herbivorous animals, thus completing the cycle. In the case of *Schistosoma* species, the cercariae are released into the water and penetrate the definitive host body through the skin (Malatji et al., 2019; Malatji et al., 2020). Fasciolosis, also known as liver fluke disease, is mainly caused by two *Fasciola* species, namely *F. gigantica* and *F. hepatica*. Even though they can infect humans, it is mainly a disease of veterinary importance in Africa. They can use snail species from the genus *Radix* (formerly known as *Lymnaea*) as intermediate hosts, but also *Pseudosuccinea columella* (Ssimbwa et al., 2014; Khanjari et al., 2014) and *Galba truncatula* (Malatji et al., 2020; Mahulu et al., 2019). Fasciolosis has a worldwide distribution and it is estimated to have the highest prevalence in the South American highlands of Bolivia and Peru (Parkinson et al., 2007; Flores C et al., 2014; Mas-Coma et al., 2020). In Africa, 11 countries were reported with a high prevalence of fasciolosis in cattle (ranging between 1.2 and 91.0%) and a lower prevalence in sheep, ranging from 0.19 to 73.7% (Khalid et al., 2017). In East Africa, Uganda was reported to have the highest prevalence of fasciolosis in cattle (70%), followed by Tanzania (38.92%), and lastly Kenya (26%) (Khalid et al., 2017). According to Joan et al. (2015), the average prevalence of fasciolosis in Uganda was 84% by 2015, causing lower milk production and an estimated annual loss of US$ 92.4 million due to condemned liver alone. Animals can be infected by schistosome blood flukes, including *Schistosoma bovis*, *S. mattheei*, and *S. curassoni*. These belong to the *S. haematobium* group species, lay eggs characterized by a terminal spine, and multiply within the *Bulinus* species of freshwater snails (Agobé et al., 2022; Joof et al., 2021). These parasites infect herbivores such as buffaloes, cattle, goats, and sheep. On the other hand, non-human primates such as baboons, monkeys, chimpanzees, and rodents can be reservoir hosts for the human *S. mansoni* species (Lindsay et al., 2019; Duplantier and Sène, 2000). Additionally, *Schistosoma* species infecting humans and animals can hybridize (Huyse et al., 2009), causing hybrid forms that can infect both humans and animals (Léger et al., 2020). This may complicate the control and elimination of schistosomiasis in areas with high livestock-wild mammal interaction, such as the Ndaiga and Kanara sub-counties in our study area.

Interventions including mass drug administration and water-based measures have been implemented in endemic areas of schistosomiasis to prevent exposure of humans to infections through contaminated water. However, fasciolosis and schistosomiasis are still persistent due to (re)infections of snail intermediate hosts, as the animal reservoir hosts are usually not targeted by most interventions (Evan Secor, 2014). Therefore, a One Health approach that considers both human health and other animal hosts (intermediate, reservoirs) must be considered to achieve better disease control. This study presents data crucial to show the prevalence of fasciolosis and schistosomiasis among wild mammals and livestock, which are often neglected in most surveys. The community livestock management practices and their knowledge about animal fasciolosis and schistosomiasis in the Kagadi and Ntoroko districts, southeast of Lake Albert in Uganda, are also presented.

## 2 Materials and methods

### 2.1 Study area

**Fig. 1:**
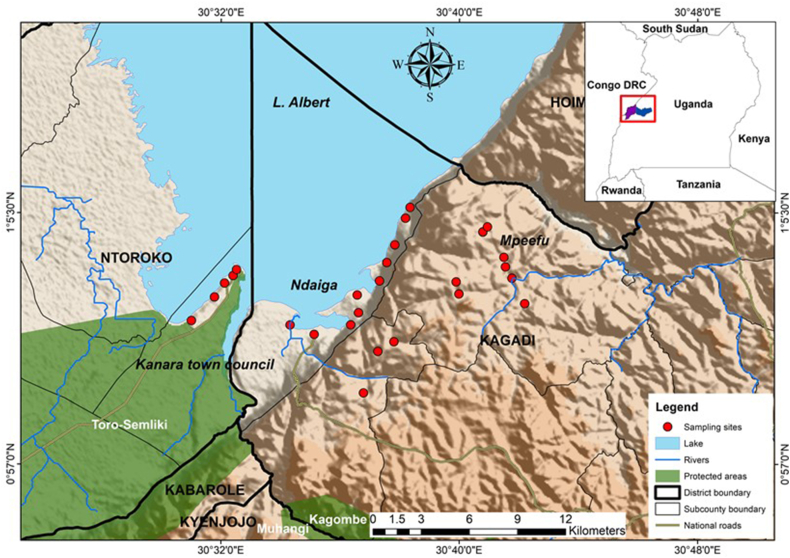
A map showing the study area(sub-counties) and sites where dung samples were collected from Kagadi and Ntoroko districts.

The study was conducted in three sub-counties of Kanara, Ndaiga, and Mpeefu located in the Ntoroko and Kagadi districts south of Lake Albert, in western Uganda. Kanara town council is located in Ntoroko district (01°06′N 30° 24′E) at an altitude of 623 m above sea level (Fig. 1). It receives an annual rainfall of 318.02 mm, and temperatures range between 17.68 and 29.03 °C per annum. The biggest portion of the Kanara town council constitutes the Toro-Semliki game reserve and the game reserve is known to be home to several wild mammals (Omeja et al., 2011). Consequently, the home range of livestock and wild mammals overlap considerably, posing a greater risk of cross-infection of parasites among animals.

Ndaiga sub-county receives 475.5 mm of rain per annum, with temperatures ranging from 13.3 to 27.46 °C. The elevations of Ndaiga and Mpeefu are 634 m and 1108 m above sea level, respectively (Fig. 1). Major economic activities in Ndaiga include fishing and livestock rearing, while Mpeefu is commercial farming and livestock rearing.

### 2.2 Ethical Considerations

This study was ethically reviewed and approved by the Mbarara University of Science and Technology Research Ethics Committee (MUST- REC). Respondents to the questionnaire consented by signing the consent forms.

### 2.3 Data collection

A cross-sectional quantitative survey was conducted from November 2019 to August 2021. It involved 12 abattoir visits and communities including those interfacing with wildlife reserves from which fresh dung samples were collected from livestock and free-ranging wild mammals. A questionnaire survey was also conducted targeting relevant key stakeholders to investigate the socio-ecological risk factors associated with the spread of *Fasciola* and *Schistosoma* spp. among livestock and wild mammals.

#### 2.3.1 Adult liver flukes and dung sample collection and examination

Twelve abattoir visits were conducted in Kagadi town and Muhorro, examining 49 cattle, 19 goats, and 10 sheep. Livers of the killed animals were examined for liver flukes by making incisions into the bile ducts, the gall bladder and adult flukes were collected and stored on 70% ethanol. In communities of Kanara, Ndaiga, and Mpeefu sub-counties, livestock herds were followed while grazing and freshly dropped dung samples were collected. Wild mammals were also followed, and dung samples were collected opportunistically from Kanara town council, bordering Toro Semliki Wildlife Reserve and Lake Albert.

The sample size for the livestock from which dung samples were collected and examined for *Fasciola* and *Schistosoma* parasite infections was calculated using the formula developed by Cochran (1965), shown below.

$$n=n=\frac{z^{2}pq}{d^{2}}$$

q=1−p

where n = the sample size; p = the proportion in the population possessing the characteristic of interest (p = 80% refer to Joan et al., 2015. While for goats and sheep p = 50%); d = the level of precision (assumed to be 5%); Z = the critical value (Z = 1.96 obtained from Z-tables).

The estimated sample size from the Cochran formula was 246, 345, and 345 for cattle, goats, and sheep, respectively, for each of the three sub-counties. However, only a total of 538, 181, and 34 dung samples from cattle, goats, and sheep, respectively, were obtained within the sampling time frame, and only samples dropped by animals at the time of collection were collected from the different sub-sites in each of the three sub-counties (Table 1).

**Table 1:** Sample size of livestock and wild mammals examined for *Fasciola* spp. and *Schistosoma* spp. eggs from Kanara, Ndaiga, and Mpeefu sub-county.

Animal category	Total (n)	Ndaiga (n)	Kaanara (n)	Mpeefu (n)
Cattle	538	257	129	152
Goat	181	72	64	45
Sheep	34	5	0	29
Hippos	62	0	62	0
Warthog	12	0	12	0
Baboons	15	0	15	0
Elephants	21	0	21	0
Monkeys	2	0	2	0

n = sample size.

A convenient sample size of fresh dung samples from free-ranging wild mammals was collected opportunistically (Lynsdale et al., 2015) from available animals within the communities of Kanara. Wild mammals from which dung samples were collected and examined for *Fasciola* and *Schistosoma* eggs included hippos, warthogs, baboons, elephants, and monkeys (Table 1).

Adult local breed livestock (both female and male) and free-ranging wild mammals were followed, and their freshly dropped dung samples were collected opportunistically and randomly. Dangerous mammals like elephants were followed at a distance while picking the dung samples. The animals from which the dung sample was collected were also noted to minimize the chances of collecting multiple samples from the same animal. For nocturnal mammals such as hippopotami, the dung samples were collected the next morning using an identification key to differentiate their dung samples from other herbivores (Chame, 2003). Only samples at a minimum of 10 m apart were considered, minimizing the chance of picking samples produced by the same animal.

Using an applicator stick 6 g of the dung sample were collected, and weighed using a digital scale (Ohaus, 2005). A new applicator stick was always used to collect a new sample to avoid cross-contamination of the samples. The samples were stored in sterile stool containers, and 10% formal saline solution was added immediately. The samples were then transported to the MUST biology laboratory for examination. The samples were concentrated using the formal-ether sedimentation technique (Zhang et al., 2012; Kaufmann, 1997) and examined for *Fasciola* and *Schistosoma* eggs using a compound light microscope (Ngwese et al., 2020; Lora Rickard, 2001). *Fasciola* and *Schistosoma* eggs were identified as species based on morphology using identification keys (Van Den Berghe, 1937; Valero et al., 2009; Graham-Brown et al., 2019; Mas-Coma et al., 2022).

#### 2.3.2 Socio-ecological risk factors

A semi-structured questionnaire was used to investigate the socio-ecological risk factors associated with the spread of *Fasciola* and *Schistosoma* spp. It consisted of 48 questions on the socio-demographics and socio-ecological risk factors, including livestock management practices, livestock-wild mammal interaction, and respondents’ knowledge about fasciolosis and schistosomiasis in animals. The questions were translated into Runyoro-Rutooro, the local dialect of the respondents in the study area.

Only key stakeholders were involved in the survey, and selected based on referrals from the local leaders. As a result, 110 key stakeholders were considered on the condition they were residents of either Kanara, Mpeefu, or Ndaiga and were 18 years of age and older. This included all veterinary officers (3), one from each sub-county, 31 butchers (5, 12, and 12), 22 herders (8, 11, and 5), 46 livestock farmers (15, 12, and 18) from Ndaiga, Kanara, and Mpeefu, respectively, and all the 8 game rangers at Toro Semliki Wildlife Reserve headquarters in Karugutu were involved.

### 2.4 Data analysis

The apparent prevalence of *Fasciola* and *Schistosoma* parasite infection was determined by expressing the number of positive animals as a percentage of the total number of animals examined (Pinilla León et al., 2019). A chi-square test was performed using SPSS version 20 at a 5% level of significance to test the difference in the prevalence of *Fasciola* and *Schistosoma* parasites in animals. The chi-square test was also performed to determine differences between management practices by respondents from different localities and to determine the associations with the socio-demographics of the respondents.

## 3 Results

### 3.1 Prevalence of *Fasciola* in livestock and free-ranging wild mammals

**Fig. 2:**
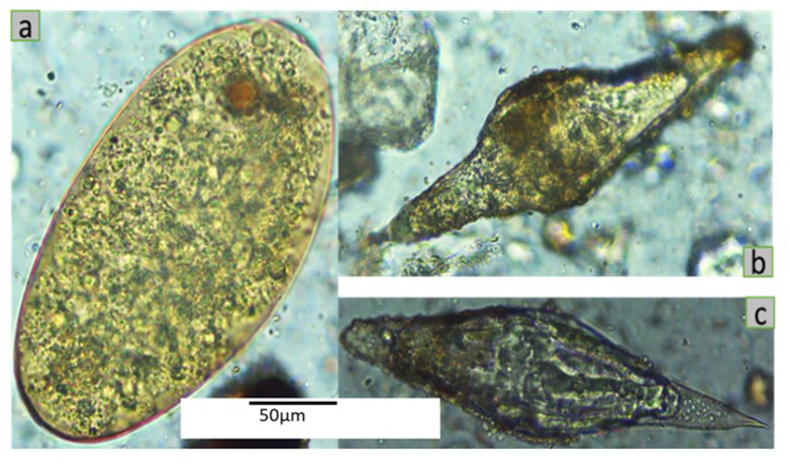
Parasite eggs recovered from cow dung samples; (a) *Fasciola gingatica* egg, (b & C) *Schistosoma bovis* eggs (magnification = 400×, scale bar = 50 μm).

In the abattoirs, 57.1%(CI 0.422–0.712) of cattle were infected with *Fasciola*, while none of the 10 and 19 examined sheep and goats, respectively, were found to be positive. Whereas 56.13% (CI 0.518–0.604), 50%(CI 0; 319-0.681), and 28.17%(CI 0.218–0.353) of the overall cattle, sheep, and goats from all sub-counties and examined based on dung and formal ether sedimentation were infected with *Fasciola* parasites. Based on the dung samples collected, the apparent prevalence of *Fasciola* species among livestock was highest at Mpeefu and Kanara (58.0% and 57.5%, respectively) and significantly lower at Ndaiga (40.4%, p < 0.001, χ^2^ = 17.969). Cattle dung collected from Mpeefu (67.1%; CI 0.59–0.745) presented the highest prevalence of *Fasciola* spp. Followed closely by that from Kanara (61.2%; 95% CI 0.523–0.69) and lowest from Ndaiga sub-county (47%; CI 0.408–0.534, p < 0.001, χ^2^ = 17.349, Fig. 2a). However, the prevalence in Kanara and Mpeefu was not significantly different (p = 0.306, χ^2^ = 1.047). Among goats, the highest prevalence of *Fasciola* spp. was obtained at Kanara (37.5%; CI 0.257–0.505), followed by Mpeefu (31.1%. CI 0.182–0.466), and significantly lower at Ndaiga (18%; CI 0.1–0.289, p = 0.037, χ^2^ = 6.585). The prevalence in Kanara and Mpeefu was not significantly different (p = 0.491, χ^2^ = 0.47). Sheep were only found in Mpeefu and Ndaiga. It was observed that more sheep from Mpeefu (51.7%, CI 0.325–0.706) were positive for *Fasciola* spp. than those from Ndaiga (33% CI 0.008–0.906; p = 0.544, χ^2^ = 0.368), see Table 2.

**Table 2:** Prevalence of *Fasciola* and *Schistosoma* spp. in livestock and wild mammals from Ndaiga, Kanara and Mpeefu sub-county.

	Ndaiga				Kanara				Mpeefu				p-value
	n	%	95% CI		n	%	95% CI		n	%	95% CI		
			lower	upper			lower	upper			lower	upper	
*Fasciola* spp.													
Cattle	257	47.1	0.408	0.534	129	61.2	0.523	0.697	152	67.1	0.590	0.745	<0.001
Goat	72	18.1	0.1	0.289	64	37.5	0.257	0.505	45	31.1	0.182	0.466	0.037
Sheep	5	33	0.008	0.906	0	0	–	–	29	51.7	0.325	0.706	0.544
Hippos	0	–	–	–	62	66	0.530	0.777	0	–	–	–	N/A
Warthog	0	–	–	–	12	8.3	0.002	0.385	0	–	–	–	N/A
Baboons	0	–	–	–	15	6.7	0.002	0.319	0	–	–	–	N/A
Elephants	0	–	–	–	21	0	0.00	0.161	0	–	–	–	N/A
Monkeys	0	–	–	–	2	0	0.00	0.842	0	–	–	–	N/A

***Schistosoma bovis***													
Cattle	257	0.43	0.022	0.075	129	0	0.00	0.028	152	2.6	0.007	0.066	N/A
Goat	72	0	0.00	0.05	64	0	0.00	0.056	45	0	0.00	0.079	N/A
Sheep	5	0	0.00	0.708	0	0	–	–	29	0	0.00	0.119	N/A
Hippos	0	0	–	–	62	0	0.00	0.058	0	0	–	–	N/A
Warthog	0	0	–	–	12	0	0.00	0.265	0	0	–	–	N/A
Baboons	0	0	–	–	15	0	0.00	0.218	0	0	–	–	N/A
Elephants	0	0	–	–	21	0	0.00	0.161	0	0	–	–	N/A
Monkeys	0	0	–	–	2	0	0.00	0.842	0	0	–	–	N/A

***Schistosoma mattheei***													
Cattle	257	0.40	0.00	0.021	129	0	0.00	0.028	152	0	0.00	0.05	N/A
Goat	72	1.4	0.00	0.075	64	0	0.00	0.056	45	0	0.00	0.079	N/A
Sheep	5	0	0.00	0.708	0	0	–	–	29	0	0.00	0.119	N/A
Hippos	0	0	–	–	62	0	0.00	0.058	0	0	–	–	N/A
Warthog	0	0	–	–	12	0	0.00	0.265	0	0	–	–	N/A
Baboons	0	0	–	–	15	0	0.00	0.218	0	0	–	–	N/A
Elephants	0	0	–	–	21	0	0.00	0.161	0	0	–	–	N/A
Monkeys	0	0	–	–	2	0	0.00	0.842	0	0	–	–	N/A

n- sample size, CI- Confidence Interval, %- prevalence percentage, N/A- Not applicable, & “–”- Not applicable.

*Fasciola* parasites were also observed among wild mammals free-ranging in the communities of Kanara sub-county sharing borders with Toro Semliki Wildlife Reserve. The *Fasciola* spp. Prevalence was highest among hippopotami samples (66%, CI 0.503–0.777), followed by warthogs (8%, CI 0.002–0.385) and baboons (6.7%, CI 0.002–0.385; p < 0.001, χ^2^ = 25.980); see Table 2. We did not record free-ranging wild animals in Mpeefu and Ndaiga.

### 3.2 Prevalence of *Schistosoma* spp. among selected livestock and free-ranging wild mammals

Generally, the observed prevalence of *Schistosoma* spp. was lower than that for *Fasciola* spp. Two species of *Schistosoma* were obtained among livestock: *S. bovis* and *S. mattheei*. *Schistosoma bovis* was only found in cattle from Ndaiga (0.43%, CI 0.022–0.075) and Mpeefu(2.6%, CI 0.007–0.066), while *S. mattheei* was found in both cattle (0.4%, CI 0.00–0.021) and goats (1.4% CI 0.00–0.075) from Ndaiga (Table 2; Fig. 2 b & c). Co-infections were also observed; for example, all livestock dung samples infected with *Schistosoma* spp. were positive for *Fasciola* spp. Therefore, 2.8% of all cattle dung samples (n = 538) had co-infection with *Fasciola* spp. and *S. bovis*. On the other hand, 0.2% of cattle and 0.6% of goats had co-infection with *Fasciola* sp. and *S. mattheei*. However, none of the wild mammal dung samples examined were positive for any *Schistosoma* spp.

### 3.3 Socio-ecological risk factors associated with the spread of fasciolosis and schistosomiasis in animals

Respondents had varied education levels, including primary (40.6%), secondary (30.2%), diploma (11.5%), no formal education (10.4%), university (5.2%), and postgraduate (2.1%). Of the total respondents, 91.9% were males, while females were only 8.1%. The socio-ecological risk factors associated with the spread of *Fasciola* and *Schistosoma* spp. were poor livestock management systems, resource sharing between livestock and wild mammals, and limited knowledge about fasciolosis and schistosomiasis by key stakeholders.

#### 3.3.1 Livestock management practices

Generally, it was observed that most respondents practiced free-range grazing on unmanaged communal land at the shores of Lake Albert in Ndaiga (100%) and Kanara (82.6%) sub-counties compared to the upland communities further away from the lake in Mpeefu (65.2%, p = 0.431, χ^2^ = 1.684). Zero grazing was reported by 21.7% and 8.7% of the respondents from Mpeefu and Kanara, respectively, and none from Ndaiga. While both paddock and tethering systems of animal rearing were the least practiced and only in Mpeefu (4.5%, Table 3).

**Table 3:** Livestock management practices reported by herdsmen and famers from Ndaiga, Kanara, and Mpeefu sub-counties.

Management practices	Location			p-value
	Ndaiga(n = 23)	Kanara (n = 23)	Mpeefu (n = 23)	
**Grazing systems**				
Free-ranging	23(100%)	19(82.6%)	15(65.2%)	0.431
Zero grazing	0	2(8.7%)	5(21.7%)	–
Paddocking	0	0	2(8.7%)	–
Tethering	0	2(8.7%)	1(4.3%)	–

**Grazing grounds**				
Communal land	19(82.6%)	22(95.7%)	15(65.2%)	0.516
Parkland	0	12(52.2%)	0	–
Lakeshores	8(34.8%)	5(21.7%)	0	–
Wetland	0	1(4.3%)	1(4.3%)	–
Private land	6(26.1%)	0	8(34.8%)	–

**Grazing ground maintenance**				
Prescribed burning	8(34.8%)	3(13.0%)	1(4.3%)	–
Controlled grazing	0	0	0	–
Enough resting time	0	1(4.3%)	0	–
Mixed grazing	0	0	5(21.7%)	–
No maintenance	15(65.2%)	19(82.6%)	17(73.9%)	0.790

**Source of water**				
Lake	17(81%)	13(61.9%)	0	–
River	4(19%)	2(9.5%)	7(31.8%)	0.189
Stream	0	0	18(81.8%)	–
Pond	2(9.5%)	4(19%)	6(27.3%)	0.329
Spring well	0	0	2(9.1%)	–
Shallow well	0	0	0	–
Wetland	0	0	1(4.5%)	–
Private water	3(14.3%)	9(42.9%)	9(40.9%)	0.087

Communal land was the most used grazing ground reported by 95.7%, 82.6%, and 65.2% of the respondents from Kanara, Ndaiga, and Mpeefu, respectively (p = 0.516, χ^2^ = 1.321). This was followed by private land, commonly used for grazing by respondents from Mpeefu (34.8%) and Ndaiga (26.1%) and none from Kanara. Lakeshores were mostly used for grazing by communities at Ndaiga (34.8%), followed by Kanara (21.7%). Many respondents (52.2%) bordering the Tooro-Semliki game reserve in Kanara reported that they grazed animals in the game reserve along with wild angulates. Lastly, wetlands were the least reported grazing sites for livestock by only respondents from Mpeefu (4.3%) and Kanara (4.3%) (Table 3).

Most of the respondents do not maintain grazing grounds, especially in Kanara (82.6%), Mpeefu (73.9%), and lastly Ndaiga (65.2%; P = 0.790, χ^2^ = 0.471). On the other hand, prescribed (also known as controlled) burning was reported by 34.8%, 13.0%, and 4.3% of the respondents from Ndaiga, Kanara, and Mpeefu, respectively. The least used form of grazing ground maintenance was “allowing enough resting time,” which was reported by 4.3% of the respondents from Kanara. However, none of the respondents reported controlled grazing, while mixed grazing was only reported in Mpeefu (21.7%; Table 3).

The most reported animal watering point, the lake, was highly reported by respondents from Ndaiga (81%) and Kanara (61.9%). Private water sources were mostly used in Kanara (42.9%) and Mpeefu (40%) and the lowest in Ndaiga (14.3%, p = 0.087). Rivers were mostly used in Mpeefu and Ndaiga (31.8% and 19%, respectively), while ponds were reported from all three three sub-counties. The use of streams, spring wells, and wetlands as animal watering points was only reported by respondents (81.8%, 9.1%, and 4.5%, respectively) from Mpeefu (Table 3).

#### 3.3.2 Livestock-wild mammal interaction

**Fig. 3:**
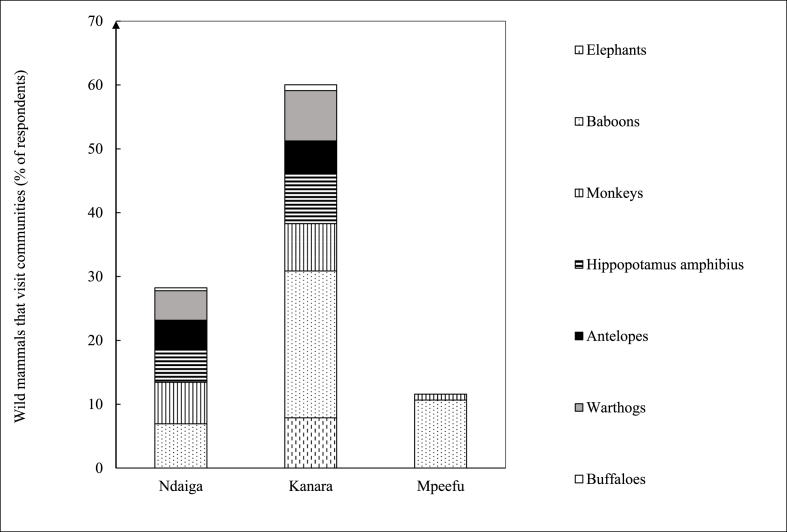
Wild mammals reported to free range in communities by respondents across the three study sub-counties of Ndaiga, Kanara and Mpeefu.

Generally, the Kanara sub-county, neighboring Toro semliki game reserve and located at the shores of Lake Albert, was reported as the most visited site by wild mammals (100%), followed by Ndaiga (90.5%), also located at the shores, and the least at Mpeefu (36.4%). Respondents from Kanara reported the highest number (6) of categories of wild mammals that visit communities, followed by Ndaiga (5) and then Mpeefu (2). The most common wild mammals that visit the communities, as reported by respondents, were baboons (89.8%), followed by monkeys (65.3%), hippopotami (57.1%), warthogs (55.1%), and lastly, elephants and buffaloes (34.7 and 6.1%, respectively). Game rangers also reported that mostly hippopotami (100%) followed by baboons (87.5%) and elephants (87.5%), warthogs (75%), and monkeys (75%), and lastly, antelopes (62.5%) and buffaloes (62.5%) commonly visit communities and can graze in the same area with livestock (Fig. 3).

### 3.4 Knowledge about fasciolosis and schistosomiasis in animals

Knowledge of animal fasciolosis among members of the three sub-counties was considerably high, ranging between 63.2 and 65.7% of the respondents from the three sub-counties (n = 110, p = 0.511). The occupation of the respondents in the animal sector showed a strong, significant association with their knowledge about fasciolosis in animals (p < 0.001, χ^2^ = 23.248, φc = 0.460). It was observed that all veterinary officers knew about fasciolosis in animals, followed by butchers (89.7%), farmers owning livestock (63%), game rangers (37.5%), and lastly, herders (31.8%). On the other hand, there was a moderate but not significant association between the respondents' education level and knowledge about fasciolosis (p = 0.072, χ^2^ = 10.119, φc = 0.303). Most of the respondents who attained university-level education (85.7%) knew about fasciolosis in animals, followed by primary level (72%), no formal education (63.6%), secondary level (50%), and lowest by diploma level (36.4%). Similarly, age was weakly and not significantly associated with the respondents’ knowledge of animal fasciolosis (p = 0.664, χ^2^ = 2.395, φc = 0.148). The age group of 58–67 had the highest proportion of respondents (77.8%) who knew about animal fasciolosis. Age groups 28–37 and 38–47 had a moderate proportion of respondents (63% and 63.6%, respectively), while 18–27 had the lowest proportion of respondents (52.8%) who knew about animal fasciolosis as shown in Table 4.

**Table 4:** Association between the knowledge of respondents about fasciolosis and schistosomiasis in animals and their social demographics.

Demographics	Knowledge about fasciolosis			Knowledge about animals' schistosomiasis		
	Aware	Unaware	p-value	Aware	Unaware	p-value
**Location**						
Ndaiga sub-county	17(65.4%)	9(34.6%)	**0.511**	2(7.7%)	24(92.3%)	**0.92**
Kanara sub-county	23(65.7%)	12(34.3%)		2(5.7%)	33(94.3%)	
Mpeefu sub-county	24(63.2%)	14(36.8%)		3(7.9%)	35(92.1%)	
**Occupation**						
Livestock owners	29(63%)	17(37%)	**0.000**	6(13%)	40(87%)	**0.000**
Herder	7(31.8%)	15(68.2%)		0(0%)	22(100%)	
Butcher	28(89.7%)	3(10.3%)		0(0%)	31(100%)	
Veterinary officers	3(100%)	0(0%)		2(66.7%)	1(33.3%)	
Game rangers	3(37.5%)	5(62.5%)		0(0%)	8(100%)	
**Education level**						
Primary	34(72%)	13(28%)	**0.072**	3(6.4%)	44(93.6%)	**0.558**
Secondary	16(50%)	16(50%)		2(6.67%)	30(93.75%)	
Diploma	4(36.4%)	7(63.6%)		2(18.2%)	9(81.8%)	
University	6(85.7)	1(14.3%)		0(0%)	7(100%)	
No education	7(63.6%)	4(36.4%)		0(0%)	11(36.4%)	
Postgraduate	2(100%)	0(0%)		0(0%)	2(100%)	
**Age**						
18–27	19(52.8%)	17(47.2%	**0.664**	1(2.8%)	35(97.2%	**0.276**
28–37	17(63%)	10(37%)		2(11.1%)	24(88.9%)	
38–47	14(63.6%)	8(36.4%)		3(13.6%)	19(86.4%)	
48–57	10(63.6%)	5(36.4%)		0(0%)	15(100%)	
58–67	8(72.7%)	3(27.3%)		0(0%)	11(100%)	

However, fewer respondents knew about schistosomiasis compared to fasciolosis in animals. Overall, 6.5% knew about animal schistosomiasis, as shown in Table 3. Similarly, respondents' occupation was strongly and significantly associated with their knowledge about animal schistosomiasis (p < 0.001, χ^2^ = 22.749, φc = 0.455; Table 4). Two out of three veterinary officers knew about animal schistosomiasis, followed by livestock owners (13%), and none of the butchers, herders, and game rangers. The respondents' education levels had a weak and no significant association with their knowledge about animal schistosomiasis (p = 0.558, χ^2^ = 3.938, φc = 0.189). Additionally, the respondent's age was weakly and not significantly associated with the respondents' knowledge of animal schistosomiasis (p = 0.276, χ^2^ = 5.108, φc = 0.215). The 38–47 age group had the highest proportion of respondents (13.6%) who knew about animal schistosomiasis, followed by the 28–37 (11.1%) and 18–27 age group (2.8%). However, no respondents belonging to 48–57 and 58–67 knew about animal schistosomiasis (Table 4).

## 4 Discussion

This study aimed to look at *Fasciola* and *Schistosoma* parasites from a One Health perspective to promote a healthy environment and healthy animals in areas with potentially high interactions between livestock and wildlife.

### 4.1 Prevalence of *Fasciola* and *Schistosoma* parasites in the study area

More cattle from the abattoirs (57.1%) were infected with *Fasciola* than those from the community (56.13%, CI 0.518–0.604). However, 0% of goats and sheep examined from the abattoir based on liver inspection had *Fasciola* parasite infection, while 28.17%, (CI 0.218–0.353) and 50% (CI 0.319–0.681), respectively, from the community were positive. Similarly, Adamu et al. (2019) reported a higher prevalence of *Fasciola* parasites, where out of the 324 cattle, 350 sheep, and 385 goats slaughtered, 38.5%, 1.14%, and 0.51%, respectively, were found positive for liver lesions of fasciolosis at Elfora Export Abattoir, compared to 33%, 1.14%, and 0.51%, respectively, obtained based on a fecal examination. *Fasciola* spp. was more prevalent than *Schistosoma* spp. in livestock and wild mammals, for example, the hippos (66% and 0%, based on dung samples). On the contrary, Kouadio et al. (2020) reported a higher prevalence of schistosomiasis in cattle, goats, and sheep killed at slaughterhouses, ranging between 5.9% and 53.3%, than fasciolosis ranging between 0 and 50.8% among livestock in farms in Côte d’Ivoire.

*Fasciola* infections were observed both at the lake shores at low altitudes (≤630m) and upland at high altitudes in Mpeefu (>1000 m). This was attributed to the wide distribution of snail intermediate host, *Radix natalensis*, that can survive in areas at altitudes <1800m (Howell et al., 2012) except in areas with very high temperatures (Stensgaard et al., 2006) in Uganda.

This study demonstrated a higher prevalence of *Fasciola gingatica* in cattle (56.1%) than what was observed in the previous studies in the Lyantonde district and the Mt Elgon regions of Uganda with prevalence values of 38.5% and 43.7% respectively (Ssimbwa et al., 2014; Howell et al., 2015). However, the prevalence recorded by this study in cattle was generally lower than that observed elsewhere in Uganda e.g, at Kampala City abattoir, which was 84% on average and 65.7% in the Lira district (Joan et al., 2015; Opio et al., 2021). This was attributed in part to the variation in the climatic variables such as temperature, rainfall, and humidity between regions, which are key in the development and hatching of *Fasciola* worms (Namutosi et al., 2019) but also may suggest the need for further comparative studies with finer resolution across the country.

The study revealed a higher prevalence of *Fasciola gingatica* among goats ranging between 18 and 37.5% than that reported in the previous studies by Zvinorova et al. (2016) at 5% and Kifle & Hiko (2011) at 4% in Zimbabwe and in Modjo in East Shawa, central Ethiopia respectively and what was reported in Sironko district located in the Eastern part of Uganda at 12% (Namutosi et al., 2019). Although Ndaiga is also located on the shores of Lake Albert, it presented the lowest prevalence of *Fasciola* in goats at 18%(CI 0.1–0.289). This was probably because farmers and herders in Ndaiga mostly graze goats on the escarpment of the Western arm of the East African rift valley with limited surface water points, thus few habitats for snail intermediate hosts and lowering their chances of grazing on pasture contaminated with the metacercariae (Zvinorova et al., 2016). Sheep dung collected from Mpeefu and Ndaiga sub-county presented a higher prevalence of *Fasciola* spp. (52%; n = 29 and 33%; n = 5 respectively) compared to 5.6% in Modjo in East Shawa, central Ethiopia as reported by Kifle and Hiko (2011), 19.4% in Northwest of Mexico(Munguía-Xóchihua et al., 2007), and 0.2% in Queen Elizabeth National park, Uganda (Nakayima and Ocaido, 2008).

The prevalence of *Fasciola* spp. was higher in cattle than in sheep and goats. This was associated with feeding habits as sheep and cattle are grazers but goats are browsers, even though farmers and herders in the study area commonly grazed the two animals together. Therefore, browsers feed on shrubs high above the ground and the chances of metacercariae attaching to them are minimal (Zvinorova et al., 2016; Kifle and Hiko, 2011). In addition, goats have the added advantage of feeding on shrubs like acacia with high tannin content, which contains an ethanolic component that is lethal to flukes (Alvarez-Mercado et al., 2015), and therefore acts as a sort of continuous deworming process. The study showed a considerable prevalence of *Fasciola* spp. among dung samples from hippopotami, baboons, and warthogs. This was in agreement with what was reported in the review by Junker et al. (2015), indicating that most wild mammals, especially herbivores, are hosts for *Fasciola* and *Schistosoma* spp. Unfortunately, we could not identify the *Fasciola* eggs to species level, as we only studied their morphology. But for the *Fasciola* species in hippopotami, we assume that it will be *Fasciola nyanzea* since this species is specific to hippos and was recently genetically confirmed in a hippopotamus from Lake Kariba by Schols et al. (2021).

From this study, the hippopotami presented the highest prevalence of *Fasciola* sp., at 66% based on dung examination. This can be explained by the fact that hippopotami are amphibious, grazing in flood plains during the night (Stears et al., 2019), which exposes them to a higher risk of ingesting grass contaminated with metacercariae (Timbuka, 2012). Similarly, *Fasciola* parasites were reported among hippopotami from Queen Elizabeth National Park (QENP) and Victoria Nile near Mackson Falls in Uganda (Thurlbeck and Lau, 1967; Leiper, 1910). Although in lower numbers, we also detected *Fasciola* spp. in dung samples from olive baboons with a prevalence of 7%. This was in agreement with Mafuyai et al. (2013) who reported that 2 out of 46 dung samples of baboons from Yankari National Park, Nigeria were positive for *Fasciola* eggs. This was attributed to the fact that during the data collection period, Lake Albert flooded and masses were displaced. Consequently, baboon habitats were also heavily affected, and given that they are generalist feeders and omnivores they were at a high risk of feeding on plant materials contaminated with *Fasciola* parasite metacercariae. About 8% of the warthog dung samples were also found positive for *Fasciola* spp., and this was attributed to them being herbivorous and feeding on contaminated vegetation in water-logged places (Cumming, 1975). However, none of the samples collected from monkeys and elephants was found positive for *Fasciola* egg and this was associated with their habit of feeding on fruits and root tubers with limited chances of metacercariae contamination for monkeys. While elephants mainly browse on shrubs that may contain ethanolic components lethal to *Fasciola* parasites (Alvarez-Mercado et al., 2015).

*Schistosoma* spp. was prevalent among livestock but not in wild mammals. Animal schistosomiasis prevalence was generally low (3%) because it's not enzootic in the region and could be from animals brought from neighboring countries (Pennance et al., 2021) and generally lower than what was reported in Northern Uganda at 37.3% by Almaz et al. (2011), and neighboring countries such as Tanzania (34%), and Ethiopia (22.2%) (Makundi and Kassuku, 1998; Gebremeskel et al., 2019) and as well what was reported in Cameroon (19%) (Djuikwo-Teukeng et al., 2019). However, this study's findings were within the same range as reported by Elelu et al. (2016) in Edu, Kwara state, northern-central Nigeria. The low prevalence was attributed to the fact that most of the dung examined was from adult animals known to develop resistance after the first infection compared to young ones between one and five years of age (Majid et al., 1980). It may also be attributed to the fact that a single dung examination that was conducted could underestimate the prevalence compared to the recommended repeated examination for at least 3 consecutive days because trematodes lay eggs intermittently and, the possibility of detecting eggs during a single dung sample examination may be minimal (Almaz et al., 2011). Additionally, most of the eggs of schistosomes get trapped in the organs and tissues hence many are not passed in the stool. Animal schistosomiasis was detected only in the sub-counties of Mpeefu and Ndaiga and not in Kanara and this was associated with the clustering of infected intermediate hosts (*Bulinus* spp) in the study sites (Gebremeskel et al., 2019). No human *Schistosoma* species were detected in any of the examined dung samples (wild mammals and livestock), unlike findings by Larbi et al. (2021) who reported that baboons and warthogs are reservoir hosts for *S. mansoni*. No *Schistosoma* species were found in sheep, which is more or less in line with Kerie and Seyoum (2016) who reported a low prevalence (2.3%) of *S. bovis* in sheep from Achefer district, northwest Ethiopia. This was attributed to sheep behavior, preferring dry environments to graze, and a distinct aversion to immersion in water (Kerie and Seyoum, 2016).

*Fasciola* and *Schistosoma* co-infections were observed in cattle and goats but not between the different *Schistosoma* species recorded in the current study. This was consistent with other previous study findings that reported a significant prevalence of co-infection between *Fasciola* and *Schistosoma* spp. reported in a review by Abruzzi and Fried (2011). In a nutshell, wild mammals and livestock can be infected by the same kind of parasites highlighting the need for integrated approaches in the control of helminths including the approaches based on the transmission cycle of the parasites and human behavior patterns in endemic areas (Lapat et al., 2024).

### 4.2 Socio-ecological risk factors

#### 4.2.1 Livestock management practices

This study revealed that most livestock owners living at the lakeshores and upland in Mpeefu practiced free-range grazing on communal land. Additionally, they used a variety of communally accessed drinking points such as lakes, wetlands, streams, and ponds which are favorable habitats for snail intermediate hosts for *Fasciola* and *Schistosoma* spp for watering animals. This explains why *Fasciola* and *Schistosoma* parasites were observed in all areas both at the lake shores with high livestock-wildlife interaction and upland where the interactions were minimal. As a result, increased interaction between livestock and wild mammals expose them to parasite populations from either groups hence increasing the risk of parasite cross infection. The observed livestock management practices were attributed to inadequate land, capital, and limited knowledge about the life cycle of the parasites that hinder farmers from practicing better animal management systems such as paddocking, rotational grazing, and provision of safe clean water, which are key approaches in controlling parasite infestation in a herd. It was also attributed to the fact that management practices at the household level in Uganda are dictated by the dependence on livestock products for income, or cultural values and food supply in association with livestock under traditional practice (Nalubwama and Mugisha, 2011) as it is in Ndaiga, Kanara, and Mpeefu The use of naturally available water sources as drinking points by farmers at livestock-wildlife interface reveals the interconnectedness between livestock and wild mammals and calls for collaborative measures between wildlife management authorities and farmers to mitigate parasite cross-transmission. The study revealed that most respondents do not involve in any activities to manage the pastures since grazing grounds are communally owned which would have helped break the parasite life cycle to ensure a parasite-free pasture land as it would work if prescribed burning is highly practiced (Kumar et al., 2013) hence their livestock are at risk of continuous reinfection from the previous seasons.

Regular deworming is recommended for livestock most especially those at the livestock-wildlife interface and those managed under traditional settings as opposed to giving treatment only when the animals show signs of sickness as reported by most respondents in this study. Deworming is key to treating the flukes in the animals, however, they are increasingly becoming resistant (Zerna et al., 2021) to some of the drugs including triclabendazole the most widely used ant-*Fasciola* drug (Fairweather et al., 2020) while in Uganda the commonly used anthelmintic drugs for livestock include albendazole, ivermectin and levamisole. It should also be noted that signs and symptoms only show when the animals have an extremely high infection load. Consequently, animals without signs and symptoms can be heavily infected and continue contaminating water bodies with the parasite eggs, thus increasing the spread of the parasites (Love and Hutchinson, 2007).

However, even though there is a higher overlap of livestock and wild mammals’ habitat, livestock from Kanara presented a lower apparent prevalence of *Fasciola* parasites than Mpeefu with no or limited livestock-wildlife interaction. This supports the notion that wild mammals can be naturally infected with *Fasciola* parasites without necessarily being a result of cross-transmission from livestock (Bargues et al., 2022). It is also true that animals sharing resources which can be parasite transmission sites can contract parasites in the fecal matter directly or indirectly from each other (Butt and Turner, 2012; Nunn and Dokey, 2006; Mafuyai et al., 2013). Hence wild mammals and livestock can be infected by the same parasites naturally and one group can be parasite reservoir host for the other. Therefore, it is important to ensure a healthy environment by advocating for inclusive interventions to control parasitic infections.

#### 4.2.2 Respondents’ knowledge about fasciolosis and schistosomiasis

It was revealed that most respondents knew about fasciolosis in livestock in their communities as opposed to schsostosomiasis in animals. This was because animal schistosomiasis is not endemic in the area except in some regions of Uganda like the north western Uganda, and areas around Lake Victoria (Stothard et al., 2004). It was also noted that respondents’ knowledge about fasciolosis and schistosomiasis in animals was significantly associated with their occupation. This implied that the likelihood of someone knowing about fasciolosis depended on their responsibility or job in animal husbandry for example most butchers knew about fasciolosis because they regularly encounter infected animals and are directly affected by the losses involved due to liver condemnation (Opio et al., 2021; Ssimbwa et al., 2014). While only veterinary officers knew about schistosomiasis in animals.

On the other hand, Knowledge of respondents about fasciolosis and schistosomiasis was not significantly associated with their education levels and age. On the contrary, Sazmand et al. (2020) reported that education, age and herd size had significant associations with knowledge groups among livestock farmers in Hamedan, Iran. This implied that to promote better public health, all individuals involved in livestock rearing regardless of their occupation, educational background, and age, it is crucial to educate them about the parasite transmission cycle most especially for zoonotic helminths as this will impact their livestock management practices. Secondly, emphasize the one health approaches by involving the local communities, healthcare authorities, and all other stakeholders, in participatory approaches to foster collaborative efforts to devise and implement inclusive control measures.

## 5 Conclusion

The study findings suggest the possibility of cross-transmission of zoonotic parasites such as *Fasciola* parasites between livestock and wild mammals. *Schistosoma bovis* and *S. mattheei* were encountered and suggested that hybridization can occur in the area upon the introduction of *S. haematobium*. However, knowledge about bovine schistosomiasis was low. Therefore, a One Health approach focusing on the integration of mass anti-helminths administration to livestock to control *Fasciola* and *Schistosoma* infections is desirable and sensitization of the key stakeholders about the parasite transmission cycle is a need in the study area. Furthermore, a molecular study to understand the zoonotic contributions of livestock and wild mammals, and confirm the cross-transmission of *Fasciola* and *Schistosoma* parasites is recommended.

## CRediT authorship contribution statement

**Daisy Namirembe:** Writing – original draft, Visualization, Methodology, Formal analysis, Data curation, Conceptualization. **Tine Huyse:** Writing – review & editing, Validation, Supervision, Methodology, Funding acquisition, Conceptualization. **Rapheal Wangalwa:** Writing – review & editing, Methodology, Formal analysis, Data curation. **Julius Tumusiime:** Writing – review & editing, Visualization, Supervision, Methodology. **Casim Umba Tolo:** Writing – review & editing, Supervision, Methodology, Data curation, Conceptualization.

## Study limitations

The main limitation of this study was a small sample size that was obtained as opposed to the proposed estimates of the animals' sample, as result this might have affected the study's power and precision of prevalence estimates.

## Funding

This study was supported by the ATRAP project (Action Towards Reducing Aquatic snail borne Parasitic Diseases) of the Belgian Development Cooperation program dd. 01.04.2014 of the Royal Museum for Central Africa (RMCA) with support of the Directorate-General.

## Conflict of interest declaration

No conflict of interest.
