# Supplementary material for: Liver fluke and schistosome cross-infection risk between livestock and wild mammals in Western Uganda, a One Health approach
Source: Int J Parasitol Parasites Wildl. 2024 Nov 19;25:101022. doi: 10.1016/j.ijppaw.2024.101022 (PMC11648790; doi:10.1016/j.ijppaw.2024.101022)
Supplement: Multimedia component 1 [file mmc1.docx]

**Social-ecological risk factor Questionnaire survey**

**A: Socio-demographic characteristics**

**Gender**

Female Male

**Sub-county………………………. Village…………………………**

**Age in years** **………**

**Marital status**

Single

Married

Separated

Divorced

Widowed

**Education status**

No education

Primary

Secondary

Diploma

University

Postgraduate

**What do you do for a living?** *(tick as many apply)*

Farmer

Fishermen

Herder

Fishmonger

Veterinary officer/ extension worker

Butcher

Business

No occupation

**B: Socioeconomic factors**

**1a) Do you have livestock?**

Yes

No

**1b) Which of these livestock do you have?** *(tick as many apply)*

Cattle

Goat

Sheep

Pigs

Poultry

All the above

**1c) How many are they?**

**Animal**  **Number**

Cattle  1-5 6-10 11-15 Above 16

Goat 1-5 6-10 11-15 Above 16

Sheep 1-5 6-10 11-15 Above 16

Pigs 1-5 6-10 11-15 Above 16

Poultry 1-5 6-10 11-15 Above 16

**1d) Which breed of animals do you have?** (you can select more than one)

Indigenous/ local breed

Exotic breed

Crossbreed

All the above

**1e) How important are the animals that you own at home?** (tick as many as apply)

Source of food (milk and meat)

Income (selling milk)

For sell (selling animal)

Ploughing garden

Paying dowery

Prestige

Cultural purposes

**Others specify**……………………………………………………………………………………

**2a) Do you normally sell some of the animals you keep at home? (if no skip to next question**

Yes

No

**If yes, which of the animals do you normally sell?** (tick as many apply)

Goats

Cows

Pigs

Sheep

Poultry

**2b) How often do you normally sell the animals?**

1. Once a year
2. Once in two years
3. After every three years
4. When I have problems/need
5. When the animal is sick
6. **Others spec**ify………………………………………………………………

**2c) Where do you normally sell the animals?** (you can select more than one)

1. Animal market
2. Butchers
3. Neighbors
4. Others specify………………………………………………………………

**C: Livestock management practices**

**3a) Which system of animal rearing do you use to rear your livestock?**

Free-range

Zero grazing

Paddocking

**3b) If free-range grazing system, which grazing grounds do you use?** (you can select more than one)

Communal land

Private land

National Parkland

Lakesides

Wetland/swamps

**3c) How many livestock owners use the same grazing ground?**

1. 1-5 cattle owners

2. 6-10 cattle owners

3. 11-15 cattle owners

4. 16 and above cattle owners

**3d) How do you maintain the grazing grounds? (**you can select more than one)

Prescribed burning

Controlled grazing

Give enough resting period

Mixed animals

**4a) What’s the source of drinking water for your livestock?** (you can select more than one)

Lake

River

Stream

Ponds

Spring well

Shallow well

Wetland

**4b) How many farmers use the same water source?**

1-5 livestock owners

6-10 livestock owners

11-15 livestock owners

16 and above livestock owners

**4c) What other activities are conducted at the water sources mentioned above that animals drink water from?** *(tick as many as applicable)*

Farming

Washing clothes

Swimming

Bathing

Playing

Laying bricks

Brewing alcohol

Fetching water

**Others specify**……………………………………………………………………

**4d) Do you think it’s safe for people and animals to share the same water sources?**

yes

No

**Give reasons for your response above**

Make water dirty and unsafe

Spread diseases

**Others specify**…………………………………………………………………………

**5a) Which problems do you face in rearing animals?** (Select as many apply)

Inadequate pasture

Cattle rustling

Diseases

Lack of water

Lack of space for rearing the animals

Drought

Flood

**Others specify**………………………………………………………………………

**6a) Which diseases often affect your animals?** (tick as many apply)

Anthrax

Brucellosis

East Coast fever

Swine fever

Fasciolosis/ liver flukes

Schsisomiasis/ bilharzia of animals

Foot and mouth disease

Footrot

**Others specify**……………………………………………………………........

**6b) How do you deal with sick animals? (***tick as many as applicable***)**

Give drugs

Isolate sick animals

Sell them

**Others specify**……………………………………………………………………

**6c) How often do you treat/ deworm your livestock?**

Once a year

Twice a year

Thrice a year

Not at all

**6dDuring which time of the year do you always treat your animals**

August September

February

April may

All times; mentioned above

None

**6e) Do you use anthelmintic drugs?**

Yes

No

**6f Which anthelmintic drug do you use to treat fasciolosis in animals?**

Triclabendazole

Albendazole

Clonsantels

Nitroxynils

Oxyclozanides

Levamisole

Ivermectin

I don’t know

**6gWhich anthelmintic drug do you use to treat schistosomiasis in animals?**

Praziquantel

Antimonials

Isothiocyanate

Trichlophone

I don’t know

**D. Knowledge and perception regarding bovine fascioliasis**

**7a) Have you ever heard of fasciolosis?**

Yes

No

**If yes, how did you know about fasciolosis? Through the;**

veterinary officer

radio

Workshop

Local leaders

**Others specify…………………………………………………**

**7b) Which of the following statements best describes the signs and symptoms of fasciolosis?**

**True False I don’t know**

1. Animal become increasingly anemic

2. Loss of appetite among the livestock

3. Pale mucous membrane of the mouth and eye

4. Animal develop bottled jaw (edema)

5. Affected animal is reluctant to travel

6. Black liver (Black disease)

7. Damaged liver with tunnels

8. Presence of liver flukes in the liver

9. Haemorrhage and fibrosis

10.Weight loss is a symptom of fasciolosis

**7c Which of the following statement best describe the effects of fasciolosis in livestock production?**

**True False I don’t Know**

1. Low productivity

2. Poor quality animal products

3. Low growth rate

4. Death of animals

5. High economic losses

**7e) People in this place think that fasciolosis in livestock is:**

**Disagree Agree Strongly agree**

1. Fasciolosis is not dangerous

2. Fasciolosis does not exist

3. Fasciolosis can be cured

4. Fasciolosis affects the liver of animals

5. People can get infected with fasciolosis

6. Fasciolosis can be transmitted from animals to human

7. Fasciolosis can be spread from humans to animals

**Knowledge and perception regarding Bovine schistosomiasis**

**8a) Have you ever heard of bovine schistosomiasis** (**Bilharzia of animals**)?

1. Yes

2. No

**If yes, how did you know about bovine schistosomiasis? Through**;

Veterinary officer

Radio

workshop

local leaders

**Others specify……………………………………………………………….**

**8b) Which of the following statements best describes signs and symptoms of Bovine schistosomiasis?**

**True False**

1. Bleeding through the nose

2. Loss of body weight

3. Swollen liver and spleen

4. Rough fur

5. Body weakness

6. Bloody or slimy feces

7. Mortality

8. Hemorrhagic diarrhea

9. Anorexia with a reduction in growth rate

10. Schistosomiasis can affect the reproductive ability of animals

**9a) Have you ever heard of bovine schistosomiasis** (**Bilharzia of animals**)?

1. Yes

2. No

**If yes, how did you know about bovine schistosomiasis? Through**;

Veterinary officer

Radio

workshop

local leaders

**Others specify………………………………………………………………………………….**

**9b) People in this area think that bovine schistosomiasis in livestock is:**

**Disagree Agree Strongly agree**

1. Goats become more severely affected than both sheep

and cattle

2. Goats and cattle develop resistance against schistosomiasis

3. Schistosomiasis naturally affect cattle, goats, sheep, and pigs

4. Schistosomiasis is spread by aquatic snails

5. the main route of transmission is the source of drinking water

6. People can get infected with bovine schistosomiasis

7. Bovinee schistosomiasis can be transmitted from animals to human

8. Bovinee Schistosomiasis can be spread from humans to animals

9. Bovinee schistosomiasis can reduce milk and meat yield of animals

10. Sharing of water sources by animals and people can expose

the animals to bovine schistosomiasis

11. Bovinee schistosomiasis is not dangerous

12.Bovinee schistosomiasis does not exist

13. Bovinee schistosomiasis can be cured

**10. Which of the following ways can be used to control fasciolosis and schistosomiasis in your community?**

**TRUE FALSE**

1. Use of strategic anthelmintic treatment to reduce the number of flukes in

the host and number of fluke eggs in the pasture

2. Improve drainage to reduce the number of intermediate host snail

3. Spray with chemicals (molluscicides) to reduce the number of intermediates

host snail

4. Rotational grazing program for the animals

5. Treating animals before moving them to a potentially contaminated area

Keeping cattle, away from water infested with snails

6. Fencing snail infested waters and grass to reduce exposure to infections

7. Remove waterweed from drinking water points for the animals

Intermittent drying of canals and troughs

**E: Interaction with wild animals**

**11a) Do wild animals often come around your community?**

Yes

No

**If yes, which of the animals often come around your community? (***tick as many applicable***)**

Warthog

Monkeys

Baboons

Antelopes

Elephants

Hippopotamus

**Others specify**…………………………………………………………………………………

**11b) Which of the following statements is true about wild animals?**

**True False**

1. Wild animals spread parasites and diseases to livestock

2. Wild animals kill livestock

3. Wild animals destroy crops

4. Wild animals are dangerous to human

**D: Impacts of floods**

**11a) During the rainy season the shores of Lake Albert flood.**

**The following are the effects of flooding on livestock animals (cattle, goat, pigs, and sheep) in your area true/ false:**

**TRUE FALSE**

1. Loss of grazing grounds

2. Increase disease susceptibility

3. Cause the death of animals

4. Displacement

**Others specify**…………………………………………………………

**11b) The people in your community do the following to protect their livestock from the impacts of floods?**

**TRUE FALSE**

1. Shift to a new area

2. Buy feeds for the livestock

3. Deworm the livestock more often

4. Sell off animals

**Others specify…………………………………………………**

**Any comment………………………………………………………....**

**You have come to the end of the survey, thank you for your cooperation**.

***END***
